# Genotyping by sequencing for estimating relative abundances of diatom taxa in mock communities

**DOI:** 10.1186/s12862-023-02104-2

**Published:** 2023-02-06

**Authors:** Ozan Çiftçi, Cornelis A. M. Wagemaker, Adrienne Mertens, Peter van Bodegom, Walter Pirovano, Barbara Gravendeel

**Affiliations:** 1 Institute of Environmental Sciences (CML), Leiden University, P.O. Box 9518, 2300 RA Leiden, The Netherlands; 2 Naturalis Biodiversity Center, Darwinweg 2, 2333 CR Leiden, The Netherlands; 3 BaseClear B.V., Sylviusweg 74, 2333 BE Leiden, The Netherlands; 4 German Research Center for Geosciences, GFZ, 14473 Potsdam, Germany; 5 Radboud Institute for Biological and Environmental Sciences, Heyendaalseweg 135, 6500 GL Nijmegen, The Netherlands; 6 Diatomella, IJkelaarstraat 3, 6611 KN Overasselt, The Netherlands

**Keywords:** Diatoms, GBS, Mock communities, Quantification, Relative abundance, Water quality assessment

## Abstract

**Background:**

Diatoms are present in all waters and are highly sensitive to pollution gradients. Therefore, they are ideal bioindicators for water quality assessment. Current indices used in these applications are based on identifying diatom species and counting their abundances using traditional light microscopy. Several molecular techniques have been developed to help automate different steps of this process, but obtaining reliable estimates of diatom community composition and species abundance remains challenging.

**Results:**

Here, we evaluated a recently developed quantification method based on Genotyping by Sequencing (GBS) for the first time in diatoms to estimate the relative abundances within a species complex. For this purpose, a reference database comprised of thousands of genomic DNA clusters was generated from cultures of *Nitzschia palea*. The sequencing reads from calibration and mock samples were mapped against this database for parallel quantification. We sequenced 25 mock diatom communities containing up to five taxa per sample in different abundances. Taxon abundances in these communities were also quantified by a diatom expert using manual counting of cells on light microscopic slides. The relative abundances of strains across mock samples were over- or under-estimated by the manual counting method, and a majority of mock samples had stronger correlations using GBS. Moreover, one previously recognized putative hybrid had the largest number of false positive detections demonstrating the limitation of the manual counting method when morphologically similar and/or phylogenetically close taxa are analyzed.

**Conclusions:**

Our results suggest that GBS is a reliable method to estimate the relative abundances of the *N. palea* taxa analyzed in this study and outperformed traditional light microscopy in terms of accuracy. GBS provides increased taxonomic resolution compared to currently available quantitative molecular approaches, and it is more scalable in the number of species that can be analyzed in a single run. Hence, this is a significant step forward in developing automated, high-throughput molecular methods specifically designed for the quantification of [diatom] communities for freshwater quality assessments.

**Supplementary Information:**

The online version contains supplementary material available at 10.1186/s12862-023-02104-2.

## Background

The increasing release of chemicals from agricultural, industrial, and domestic sources in the last few decades has led to significant contamination of aquatic ecosystems. The freshwater compartment is particularly vulnerable to such anthropogenic impacts, and large-scale monitoring programs have been established to assess the resulting degradation, such as the Water Framework Directive (WFD) [1]. Biofilms (communities of organisms attached to surfaces) are one of the biological compartments recognized by the WFD as a target for freshwater quality assessment due to their rapid responses to environmental changes, rapid growth rates, and physiological variety of the constituent organisms [2]. Microalgae are the dominant members of these communities and have a vital role as primary producers. They are sensitive to many environmental variables (e.g., salinity, pH, nutrient concentrations) and have traditionally been used to classify water bodies based on autecological preferences of the community’s taxonomic composition [3].

Gathering information on microalgae community composition and abundance is, however, a challenging task that requires taxonomic expertise and specialized tools. These challenges apply particularly to diatoms, a group of microalgae that are present in all types of waters that are highly sensitive to eutrophication/organic pollution gradients [4]. Conventionally, diatom taxonomists identify and count several hundred diatom valves in biofilm samples using light microscopy. Specialized tools for microalgae have also been developed which integrate sampling devices, image analysis technologies, and machine learning algorithms, such as ZOOSCAN [5], VPR [6], and FlowCam [7]. However, these tools provide limited taxonomic resolution when morphological differences are subtle. Moreover, recent molecular phylogenetic studies have shown that many diatom morphospecies comprise several evolutionary lineages likely corresponding to species-level differentiation [8–11]. It is essential to recognize these ‘cryptic’ species because their ecological niches may differ even when they live in sympatry [12, 13], or when different localities harbor different proportions of morphs with varying ecological tolerances [14]. Moreover, indices based on diatom community metrics that are currently in use for freshwater quality monitoring in European countries [15–17] require the identification and quantification of hundreds of species which include morphologically similar and/or phylogenetically close taxa.

Molecular methods can overcome some of these challenges. For example, qPCR and ddPCR methods have been developed to assess the abundance and distribution of sub-populations of plankton in environmental samples [13, 18–20]. However, these methods require a priori information on the target gene of the focal populations and are limited to surveys targeting certain species or genera, hampering scalability which is crucial for environmental assessments. DNA metabarcoding can overcome this issue, and several studies comparing metabarcoding with microscopy methods have been published in the last decade [10, 21–27]. In general, metabarcoding has proven to be a valuable tool for detecting rare species and overall changes in community composition. However, several issues have been highlighted for obtaining reliable abundance estimates, including; (1) reference database incompleteness, (2) lack of resolution of phylogenetic markers, (3) cryptic diversity, and (4) gene copy number variation [28, 29]. Moreover, the correlations of gene copy numbers and genome sizes with biovolumes of different species need to be considered for reliable estimates [13]. Therefore, morphological assessment remains to play a central role despite the many advantages offered by these more recently developed molecular approaches [30–32]. There are several additional High Throughput Sequencing (HTS) based methods that have been used in recent years to quantify species abundances in plant mixtures, including genome skimming and multispecies genotyping by sequencing (msGBS) [33–35]. These methods could have great value in applications that rely on the identification and quantification of microalgae in environmental samples, such as freshwater quality assessments because they are scalable in species numbers (i.e., more species can be analyzed in a single sequencing run depending on the sequencing depth) and provide a taxonomic resolution comparable to the surveys performed by taxonomists.

*Nitzschia palea* is one of those widespread bioindicator species complexes with several morphological variants described from either organic- and metal-polluted or clean and only slightly polluted habitats [2, 17, 36, 37]. Morphological differences between these varieties are very subtle, and their differentiation using light microscopy is impossible [38]. Molecular data (*rbc*L, 28S rRNA, and *cox*1 genes) suggest no objective criteria to choose a precise molecular threshold for species boundaries in *N. palea*, although the complex does contain several lineages [12, 39, 40]. Ciftci et al. [41] revealed three evolutionary lineages based on 183 genes and detected recent gene flow between clades with different morphologies and a resulting putative hybrid. It is important to identify and quantify the abundances of these intraspecific lineages to reveal any differences in their distribution and ecology.

In this study, we aimed to evaluate a genome-based quantification method, msGBS, to estimate the relative abundances within the *N. palea* species complex. msGBS is based on randomly fragmenting genomic DNA (i.e., nuclear, mitochondrial, and plastid DNA) using endonucleases, and amplifying these fragments with ligated synthetic adapters. Therefore, only a subset of the genome is sequenced providing a middle ground between targeted and whole genome sequencing [35]. The sequencing reads originating from each monoclonal sample are clustered into a relatively small reference genome, and the reads originating from mixed samples are mapped against these reference clusters. Homologous clusters among taxa are removed from the reference providing increased taxonomic resolution, and the high-throughput sequencing allows analyzing many taxa in a single run providing increased scalability. Moreover, a calibration key is generated from samples of equal cell proportions (i.e., calibration samples) to convert read counts to taxa abundances, which corrects for biases related to variations in typical DNA yields from different taxa. We used msGBS on mock mixtures prepared from six strains belonging to three *N. palea* lineages for (1) resolving closely related taxa within a non-model diatom species, and (2) comparing quantification accuracy with traditional light microscopic surveys.

## Results

### GBS library preparation and clustering

A total of 36 DNA isolations were performed with concentrations ranging from 5 to 82 ng/µl. DNA yield for the pooled sequencing library was 1.66 ng/µl, and the average fragment size was 1003 base pairs. We obtained 113,369,922 reads from this library, 56.7% containing adapter barcodes. All retained reads after demultiplexing passed quality filtering, and the number of reads per sample ranged from 48,354 to 6,638,094. Around 50% of the reads were identified as duplicate reads. After dereplication, 32,114,904 read pairs were retained, of which 14.2% were merged, and 85.8% were joined.

We obtained a low number of reads for one monoclonal sample (TCC13903) due to failed library preparation. Therefore, we removed this strain from the meta-reference and repeated the data analysis starting from the mapping step. We also excluded the valves assigned to TCC13903 from the light microscopy dataset for comparison. Thus, the total number of valves in the final LM dataset was lower than 200 for a majority of the mock samples. Similarly, we recalculated the relative abundances of mock preparations (i.e., expected values) after subtracting the number of cells included from TCC13903.

The number of total clusters that we obtained after the removal of TCC13903 and the clusters that passed all filtering steps are listed in Table 1. TCC907 had the lowest average number of reads per cluster in the meta-reference, probably due to the higher genetic polymorphism of its allopolyploid genome [41].

**Table 1:** Summary of sequence clustering and filtering steps during meta-reference construction

Strain	Total number of clusters	Number of clusters after filtering	Number of reads after filtering
TCC13901	10,503	6243 (59.4%)	992,811
TCC523	13,440	8877 (66.0%)	1,662,267
TCC641	24,240	13,710 (56.6%)	1,752,771
TCC852	14,986	5285 (35.3%)	1,104,154
TCC907	14,649	13,332 (91.0%)	359,650

Among the clusters filtered based on homology, non-target mappings were most common between TCC523, TCC641, and TCC852 (Table 2, Additional file 1: Table S1), the members of a single clade based on the 183 nuclear gene phylogenies provided by Ciftci et al. [41]. BLASTN filtering removed 1.48% of the clusters in the meta-reference. After filtering, 98.7–99.8% of the reads were mapped to the target monoculture sample, and a total of 47,447 clusters were retained in the final meta-reference database.

**Table 2:** The number of gDNA clusters filtered due to homology

	TCC13901	TCC523	TCC641	TCC852	TCC907
TCC13901	2733^*^	172	485	390	480
TCC523	12	1716*	1421^2^	1173^4^	241
TCC641	186	169	8677^*^	1308^3^	190
TCC852	900	212	5623^1^	2278^*^	688
TCC907	206	0	39	20	1052^*^

*Target mappings

^1,2,3,4^Non-target mappings of more than 1000 clusters (ordered)

One calibration sample out of five also gave a low number of reads, and a four-sample calibration set was used in the subsequent steps. We obtained 267,307–305,356 reads from the calibration samples. The distribution of reads within calibration samples varied largely among strains (4596–151,562). However, the variance of the relative read counts across calibration samples was low (s^2^ between 0.04 and 0.74). Two mock samples (1 and 11) were removed from the GBS dataset due to a low number of reads after cluster filtering (< 3000) (Additional file 1: Table S1). Among the 23 samples retained, one (sample 21) had a slightly higher number of reads than the filtering threshold (3653 reads), and the remaining 22 samples had 234,602 to 2,497,370 reads (Additional file 1: Table S1). In total, 22,494,098 read mapping events were registered to 34,933 clusters in the meta-reference (i.e. 73.6% of the total number of clusters), including monoculture, mock, and calibration samples.

### False positive and false negative detections

Light microscopy (LM) counts distinguished strains based on their length, width, and fibula density features (Additional file 1: Table S2). These features overlapped with the morphometric ranges provided by Çiftçi et al. [41], except for TCC907 (Additional file 1: Table S3). This strain is reported to be a recent putative hybrid, and it had many morphological deformities due to culture conditions. Among the six strains analyzed in this study, the highest number of false positives using LM was for TCC907, which was falsely detected in five mock samples (Table 3). False-positive detections in mock mixtures were more common with GBS than LM (Table 3). However, the falsely detected strains with GBS had the lowest cell count in the respective mock sample for most of these cases, indicating that the GBS method tends to assign a small proportion of reads to other strains. The only false-negative detection was for mock sample 22, where TCC523 and TCC852 were missed in LM counts.

**Table 3:** The number of false positive (FP) and false negative (FN) detections using LM and GBS methods

Strain	LM		GBS	
	FP	FN	FP	FN
TCC13901	4	0	7	0
TCC523	3	1	7	0
TCC641	2	0	6	0
TCC852	4	1	6	0
TCC907	5	0	7	0

### Relative abundance estimates across strains

**Fig. 1:**
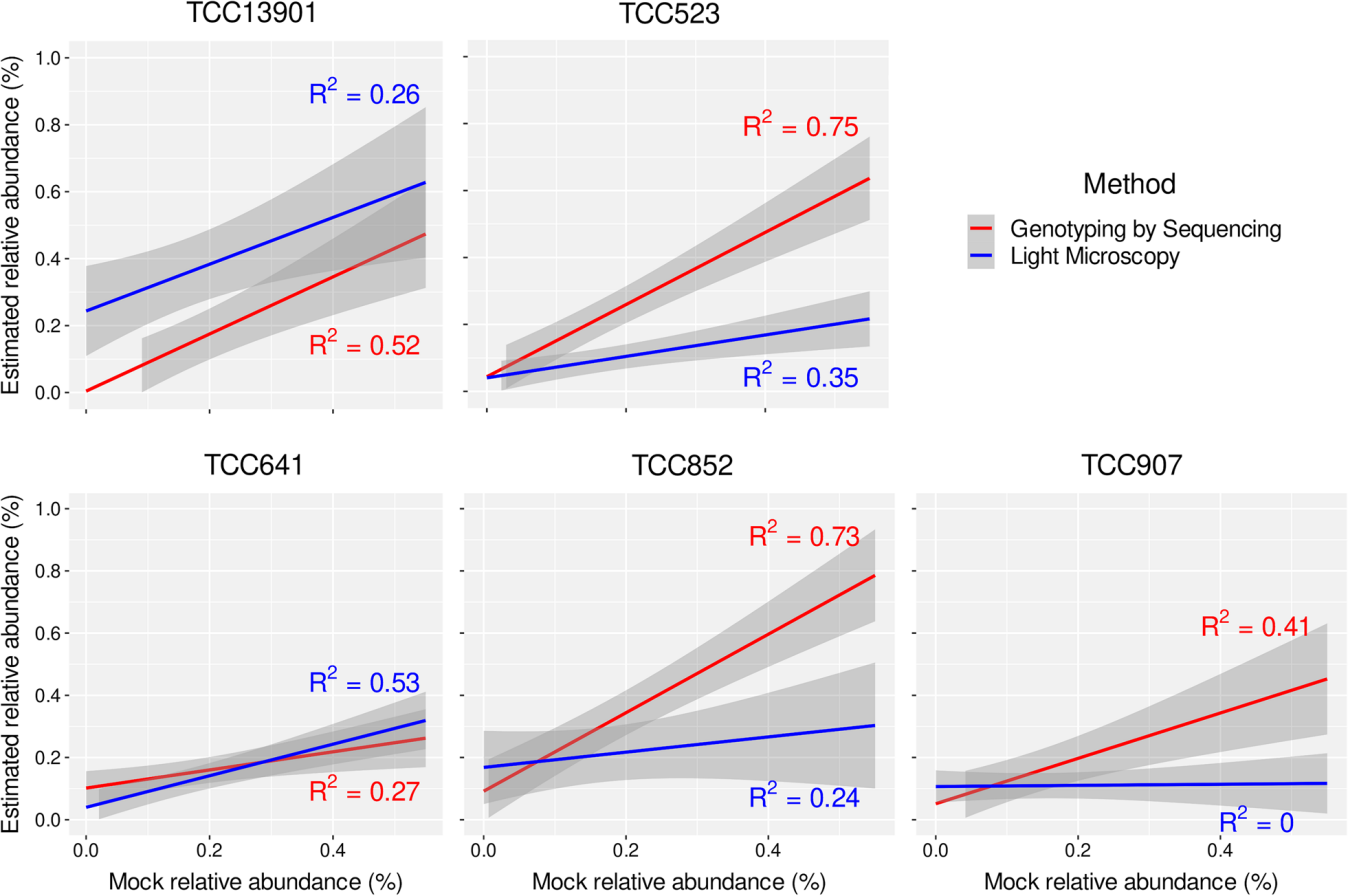
Regression lines (blue and red) and coefficients of determination for relative abundance estimates per *Nitzschia palea* strain across mock samples. 95% confidence intervals (grey) are drawn around the regression lines

We obtained moderate-to-strong correlations (R^2^ > 0.5) for three out of five strains (TCC13901, TCC523, and TCC852) when GBS estimates across mock samples were compared with microscope counts, whereas only TCC641 had a moderate correlation with LM estimates (Fig. 1). Moreover, the GBS-based method over-estimated the mock relative abundance of one monoclonal sample (TCC852) and under-estimated another (TCC641), whereas LM over- or under-estimated all. Confidence intervals were narrower for GBS estimates indicating that GBS was more accurate than LM. The removal of the monoculture sample TCC13903 from the dataset had a slightly positive effect only for one strain (TCC523), while the correlations for TCC641 decreased significantly (from 0.46 to 0.27).

### Relative abundance estimates across mock samples

**Fig. 2:**
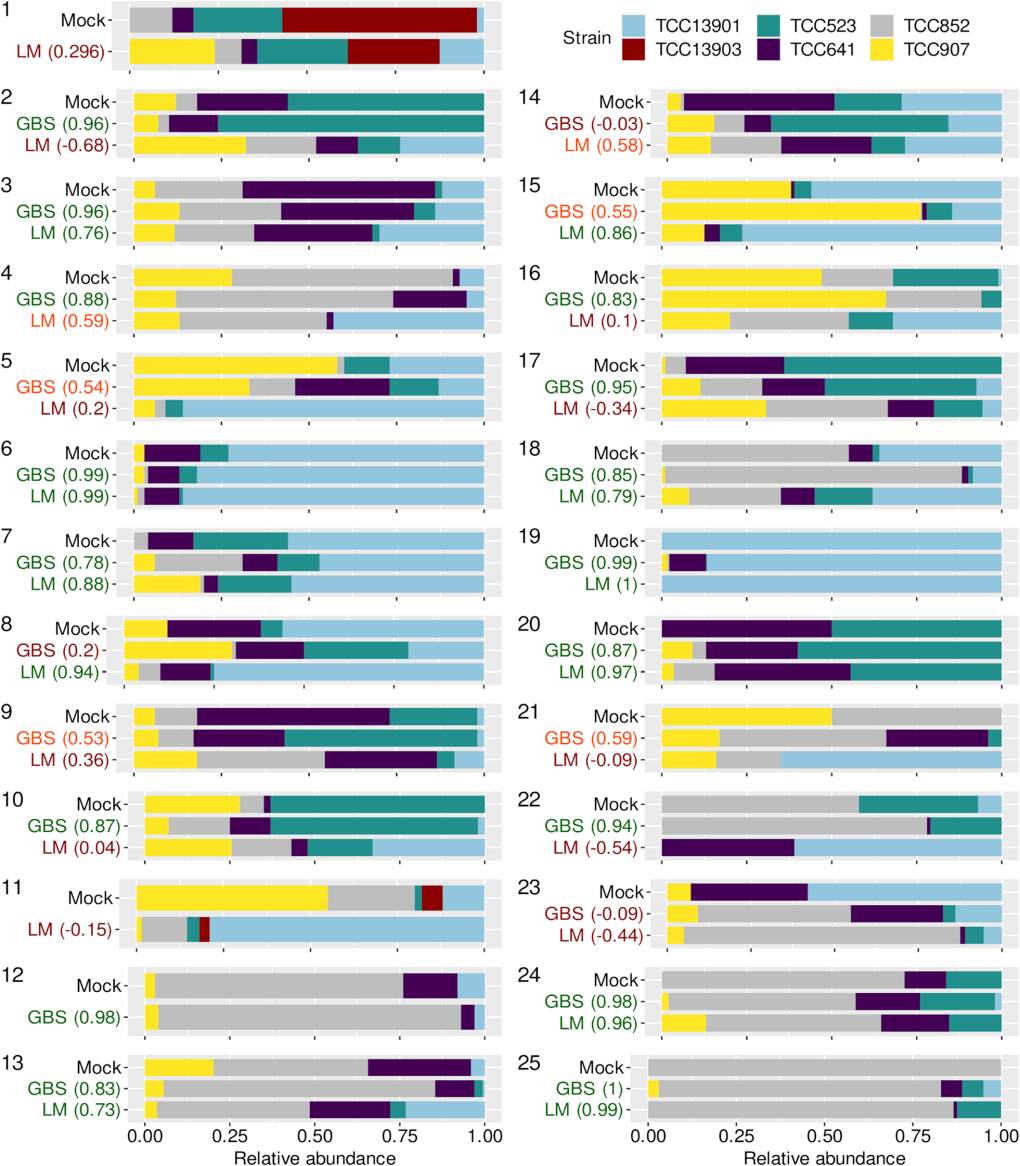
Composition and relative abundance estimates of mock *Nitzschia palea* samples obtained by using Genotyping by Sequencing (GBS) and Light Microscopy (LM) methods. Correlation coefficients were calculated using the Pearson correlation test. The green text indicates a strong correlation (> 0.7), the orange text indicates a moderate correlation (0.5–0.7), and the red text indicates a weak or no correlation (< 0.5)

Out of the 22 mock samples analyzed using GBS and LM, relative abundances for nine samples had strong correlations (> 0.7) using both methods (Fig. 2). A higher degree of correlation was obtained using GBS in 15 of these samples, whereas LM performed better in six samples. Only sample 23 had weak correlations with both methods. Two samples, 1 and 11, were analyzed using only LM and these had weak or no correlations with mock relative abundances. Sample 12 was analyzed only with GBS, and the correlation was very strong (0.98) for this sample. For mock samples 19 and 25, which contained a single *N. palea* strain, 14% and 20% of the reads were mapped to other strains, respectively. Similarly, on average, 6% of the reads mapped to absent strains in mock samples (false positive signal). Out of 17 cases of false positive detections of a single strain with both methods, only three had stronger signals (i.e., a higher proportion of reads) with GBS, indicating that the impact of false positives on GBS-based relative abundance estimates is lower than LM. The only mock sample with a low number of reads that we included in the analysis, sample 21, had a moderate correlation with GBS, demonstrating that sequencing depth can influence GBS-based estimations.

## Discussion

This study aimed to evaluate msGBS to estimate the relative abundances within the *N. palea* species complex, as a possible first step toward developing a quantitative, high-throughput molecular approach for the quantification of diatom communities for freshwater quality assessments. The varieties described from waters with different pollution levels (*N. palea* and *N. palea* var. *debilis*) have considerable overlap in their morphological characteristics, making the species complex taxonomically challenging [12, 38, 40]. Ciftci et al. [41] recovered three strains analyzed in this study (TCC523, TCC641, and TCC852) in one molecular clade with lanceolate morphology. In contrast, two of the remaining three strains (TCC13901 and TCC13903) had narrower linear-lanceolate morphology. It has been demonstrated that there is a recent gene flow between these clades with different valve outline characteristics and a resulting putative hybrid (TCC907). Understanding the distribution and abundance of intraspecific lineages in these taxonomically challenging species might be important if they have distinct habitat preferences due to ecological affinities associated with varying environmental conditions, as demonstrated for other *Nitzschia* species [42]. For this purpose, we evaluated msGBS compared to conventional light microscopy counts to identify and quantify six *N. palea* strains in mock mixtures.

### GBS-based relative abundance estimates show stronger correlations than LM with mock abundances

The morphometric ranges measured with LM overlapped for three strains that were morphologically and phylogenetically closest (TCC523, TCC641, and TCC852) [41]. These were smaller than the ranges indicated for the species due to size reduction in long-term cultures [43], and some of their valves had deformities due to culture conditions. Therefore, it was challenging to distinguish these strains using LM, and their mock relative abundances were underestimated with weak to moderate correlations. GBS-based estimates were more strongly correlated with the mock abundances for two of these morphologically similar strains (TCC523 and TCC852), and LM performed better for TCC641. Also, the relationship between GBS-based estimates and mock abundances did not follow a close relationship to the ideal 1:1 ratio for these three strains. The relative abundance of TCC641 was under-estimated, whereas TCC523 and TCC852 were over-estimated. This might be due to their close phylogenetic relationship which would cause incorrect mapping of the reads originating from TCC641 to the reference clusters of the other two strains. However, we obtained a stronger correlation for TCC641 before removing TCC13903 from the GBS dataset, indicating that better estimates could be obtained with the original dataset. The number of clusters removed due to non-target mappings between pairs of monoclonal samples was also higher for TCC523, TCC641, and TCC852 (Table 2, Additional file 1: Table S1), confirming their close phylogenetic relationship. Nevertheless, we obtained stronger correlations using msGBS for four out of five strains, highlighting that molecular methods should be used to capture the underlying phylogeny when morphologically similar taxa are analyzed.

TCC13901 is a distinct lineage with the highest stria density among the strains analyzed in this study. However, striae are usually not visible with LM in *N. palea.* Therefore, this distinguishing feature could not be examined, and the valves originating from other strains were probably assigned to TCC13901 with LM, causing an overestimation of its relative abundance. The correlation of GBS-based estimates with the mock abundances for TCC13901 was stronger than LM, and its regression slope was closer to the ideal 1:1 ratio, indicating a higher precision.

GBS-based estimates were more strongly correlated than LM with mock abundances for a higher proportion of the mock samples. However, on average, 6% percent of the reads were assigned to absent strains in the msGBS setup, creating a low but persistent false positive signal (Table 3). This signal did not significantly impact the relative abundance estimates as we obtained strong correlations. However, this issue and a large number of sequence clusters mapping to phylogenetically similar taxa highlight that genetic variation among the populations of the target diatom species should be considered for the msGBS design. It might be possible to further minimize this impact by changing the parameters in the data analysis pipeline or by introducing additional steps.

### GBS provides sufficient resolution for the detection of intraspecific hybrids

In the analyses for the study presented here, the putative hybrid (TCC907) was the most falsely detected strain using LM. Moreover, we obtained no correlation between mock relative abundances and microscope counts for TCC907. Part of the problem with the morphological identification of TCC907 might be the dominance of teratological forms in the cultures of this strain. Additionally, identification from valves can get complicated in the case of hybrids, as demonstrated for other diatom species [44–46]. GBS-based relative abundance estimates, on the other hand, were moderately correlated with the mock abundances for TCC907, indicating that thousands of gDNA clusters generated from a monoclonal culture can provide the necessary phylogenetic resolution to detect intraspecific hybrids. Sequencing depth can also be an important factor in this sense, as deep sequencing would provide a larger number of unique clusters and increase resolution.

### The calibration procedure prevents biases related to gene copy number variation

The number of reads in our calibration samples varied up to 32-fold among strains but were consistent across samples indicating strain-specific biases in the final sequencing library (Additional file 1: Table S1). Variations in the DNA contents per cell of these strains might explain this difference. However, little is known about the DNA content per cell for diatoms. It has been suggested that there is a proportional relationship between cell size and DNA content in algae and eukaryotes in general [47, 48]. However, the highest proportion of reads in both monoclonal and calibration samples originated from the strain with the smallest cells in our sample set (TCC523, Additional file 1: Table S1 and S3). Therefore, the variation of the number of reads in calibration samples might be introduced during sample processing (e.g., DNA extraction or library preparation) or originate from gene copy number variation. Either way, the impact of these biases is minimized in msGBS, because the number of reads in mock samples for each strain is calibrated based on the relative number of reads obtained from these calibration samples.

### msGBS compared to current quantitative molecular approaches

Previous studies compared species-specific qPCR assays with LM to detect and quantify the relative abundances of populations of *Navicula phyllepta* [19] and *Pseudo-nitzschia pungens* [13]. nrITS genotypes analyzed in these studies had subtle consistent morphological differences, but identification was problematic with LM in both cases. ddPCR methods have also been used in quantitative studies and proved to be more precise and accurate than qPCR [20, 49]. However, both qPCR and ddPCR methods rely on species-specific primers that can only amplify a few selected genes that span a few thousand base pairs combined. Therefore, these methods provide limited scalability and phylogenetic resolution. gDNA clusters generated in this study for the meta-reference, on the other hand, included more than 5,000,000 reads with an average length of 150 base pairs from both coding and non-coding regions of nuclear, plastid, and mitochondrial genomes. Moreover, the unique clusters of each monoclonal sample were retained during the meta-reference construction and a calibration key is used to convert read counts to relative abundances. These features allowed the GBS-based method to eliminate some critical drawbacks of the currently used quantitative molecular approaches [28, 50], as (1) monoclonal samples can be identified by taxonomists resulting in a more robust and locally representative reference database compared to publicly available data, (2) thousands of gDNA clusters originating from different cell compartments provide sufficient taxonomic resolution for strain-level identification*,* and (3) calibration samples eliminate the biases introduced due to factors such as variations in gene copy numbers or DNA content. Nevertheless, using msGBS on different groups of organisms would require optimization of the methods. Therefore, qPCR/ddPCR methods are still more practical for small-scale quantitative experiments. Metabarcoding, on the other hand, is not developed for quantitative assessments and recent review studies suggest that additional research is required to reliably use it in quantitative applications [29, 50] (see Table 4 for further comparisons).

**Table 4:** Comparison of msGBS to currently available molecular methods for species identification and quantification

	msGBS	qPCR/ddPCR	Metabarcoding
Quantitative information	Quantitative through the use of calibration samples	Fully quantitative through standard curves	Possibly semiquantitative (further research is required)
Scalability (number of species)	High (depending on sequencing depth)	Low (i.e., only a few species)	High (depending on sequencing depth)
Sensitivity (i.e., detection capability)	Further research is required on field samples	High—very high, (i.e., depends on the primer efficiency)	High (i.e., sometimes lower than qPCR/ddPCR)
Taxonomic resolution	Subspecies, variety (i.e., depends on the number of reference clusters)	Species-level or above (i.e., might be difficult or impossible for very closely related species)	Species-level or above (i.e., might be difficult or impossible for very closely related species)
Laboratory work and data analysis	Requires highly trained personnel	Relatively easy owing to standardized instruments and software	Requires highly trained personnel

### Implications of msGBS for biomonitoring

The next step would be to develop and test GBS for field samples collected from freshwater habitats. Theoretically, the relative abundances of the most common diatom species in a given water body or system can be estimated for hundreds of environmental samples in a single msGBS analysis, allowing simultaneous calculation of diatom-based biotic indices. Previous studies on relative abundance estimation of diatoms highlighted several problematic species that cause significant abundance discrepancies, including *N. palea* [24, 32, 50]. In another recent example, significant relative abundance discrepancies due to gene copy number variation were detected when calculating a commonly used index for benthic diatoms from DNA metabarcoding data of the *rbc*L gene [28]. Taxonomy-free approaches based on OTUs are also available and infer a molecular index directly from sequencing data [28, 51, 52]. In contrast, msGBS relies on thousands of gDNA clusters that provide an improved taxonomic resolution, and higher precision in quantification than light microscopy counts. Calibration samples correct for biases related to variations in typical DNA yields between taxa. Therefore, a single calibration key can be used for a given set of taxa in msGBS and it is not necessary to include calibration samples in further sequencing runs. As the msGBS setup is highly scalable in terms of the number of species that can be analyzed in a single run, it has great potential in calculating indices based on benthic diatoms and developing automated quantification methods for microalgae in general. One of the critical drawbacks of msGBS is its reliance on monoclonal cultures which are not easy to obtain and maintain for diatom species. Culture collections are critical resources in this sense as the application of such new high-throughput methods will require sampling additional genomic loci from different diatom species. In this regard, it is important to note that preserved DNA extracts are equally valuable for applications such as msGBS because the use of the same restriction enzymes, sequencing depth, and clustering parameters is expected to result in identical reference data. Although msGBS has already been tested on field samples for plant roots [35], it is also necessary to further test it for diatoms before confidently utilizing it.

## Conclusions

Our evaluation of msGBS shows that it can resolve closely related lineages within a non-model diatom species and provides improved precision compared to conventional light microscopic surveys. However, we detected a low and persistent false positive signal which suggests that the genetic variation among the populations of the target diatom species should be considered to obtain accurate relative abundance estimates when phylogenetically close taxa are studied. Nevertheless, msGBS performed better than LM counting for identifying and quantifying a putative hybrid, indicating that it provides unprecedented resolution compared to surveys targeting a few phylogenetic markers. In this sense, we highlight sequencing depth as a critical factor because phylogenetic resolution depends on the number of unique clusters in the meta-reference, and deeper sequencing would further increase the resolution. The calibration procedure minimizes the impacts of biases related to variations in gene copy numbers or DNA contents, which is another critical advantage of msGBS over the currently used molecular approaches. Therefore, we suggest that genome-based HTS approaches, such as msGBS, can have significant implications for the quantification of microalgae.

## Methods

### Algal cultivation and preparation of mock communities

We acquired six *N. palea* strains from Thonon Culture Collection, France (TCC) [53] (Table 5). The cultures were grown in WC medium [54] at 19 °C and on a 16 h light/8 h dark cycle. We routinely examined the live cultures under a Zeiss Axio Imager M2 microscope and transferred the cells every 1–2 weeks based on the observed growth rates of individual cultures. Several harvests from each strain were collected at their exponential growth. These cells were concentrated in 2 ml tubes and counted using a Zeiss Axio Imager M2 microscope under brightfield (DIC) and a Neubauer counting chamber (Carl Roth, Germany). Three replicates were counted for each cell suspension (Additional file 1: Table S4), and we harvested additional cultures until a minimum of 6 million cells per strain had been collected. Suspensions with concentrations above or below the recommended ranges for counting with a Neubauer counting chamber (i.e., 250,000–2.5 million cells/ml) were either diluted in dH_2_O or concentrated by centrifugation. Finally, we prepared 25 mock samples by mixing the volumes from each suspension that contained the required number of cells for the GBS setup (Additional file 1: Table S5). In the mock sample set, 18 samples were mixes of five strains, three were mixes of three strains, three were mixes of two trains, and one contained a single strain. Mock samples contained an estimated number of one million cells in total. In addition to these, we prepared five calibration samples that contained an estimated number of 990,000 cells in equal proportions from each strain (Additional file 1: Table S6). We used a much larger number of cells for monoclonal samples because we needed a sufficient number of reads and unique clusters in the meta-reference database. Therefore, the number of cells in monoclonal samples was not estimated by counting. All samples were either diluted or concentrated to a final volume of 2 ml. Finally, we aliquoted 100 µl from mock samples containing, an estimated 50,000 cells, for light microscopy counts by a diatom expert.

**Table 5:** Collection and isolation information of *Nitzschia palea* strains acquired from the Thonon Culture Collection (TCC) and sequenced in this study

Strain identifier	Collection strain identifier	Locality	Isolation date
TCC13901	TCC139-1	Lake of Geneva, France	04/11/2009
TCC13903	TCC139-3	Lake of Geneva, France	03/12/2010
TCC523	TCC523	River, Saint-Denis, La Réunion	10/02/2010
TCC641	TCC641	River, Viichtbach, Boevange/Attert, Luxembourg	27/01/2010
TCC852	TCC852	Upland stream, Casal da Misarela, Portugal	10/04/2013
TCC907	TCC907	River, Northumberland, United Kingdom	01/01/2015

### Light microscopy slide preparations and counts

We transferred the 100 µl aliquotes from each mock sample to glass tubes and oxidized these samples with hydrogen peroxide on a heat block for 30 min at 90 °C following Handboek Hydrobiologie [55]. The oxidized samples were washed twice with distilled water (centrifugation for 5 min at 4000 g) and dissolved in 100 µl distilled water. Two slides were prepared per mock sample using Naphrax^®^ as the mountant, and the slides with the better spread were selected for light microscopy analysis on a Zeiss Axioskop 40 using phase contrast with a magnification of 1000x (n.a. 1,30). In total, 200 valves per mock sample were measured and identified per microscope slide.

### DNA extractions

Genomic DNA was extracted manually from all samples using a modified CTAB extraction procedure. In total, 36 samples were used, including 25 mocks, five calibrations, and six monoclonal samples. Monoclonal samples were harvested by removing the excess medium from 100 ml cultures and concentrating the cells in 2 ml with repeated centrifugation steps for 10 min at 4000 g (4 °C). Calibration and mock samples were prepared in 2 ml volumes from these concentrated samples. All samples were initially concentrated to 50 µl through repeated centrifugation steps (10 min., 15,000 g, 4 °C). Final suspensions were transferred to tubes containing 700 µl of CTAB lysis buffer (BioChemica) mixed with 10 µl of beta-mercaptoethanol (Sigma-Aldrich) and 20 mg/µL of RNAseA (Sigma-Aldrich) and pre-soaked with 0.5 ml of zirconia/silica beads (0.5 mm, BioSpec). Bead beating was performed using a Qiagen Tissue Lyser II for 5 min, and the lysates were incubated at 65 °C for 45 min with shaking every 5 min. DNA was purified using 700 µl of Chlorophorm-Isoamyl Alcohol (24:1), and the upper phase was recovered after centrifugation (10 min, 15,000 g, 4 °C). This recovered phase was mixed with a double volume of cold 96% Ethanol and 225 µl NaOAc, and incubated at − 20 °C for 1 h. DNA was precipitated through centrifugation for 15 min at 15,000 g (4 °C), cleaned with cold 70% ethanol, and the dried pellets were dissolved in 30 µL double-distilled H_2_O. DNA concentrations and purity was controlled on a DropSense96 (Trinean) System.

### GBS library preparation and sequencing

The GBS protocol and sequencing followed Wagemaker et al. [35] with minor modifications. In brief, extracted genomic DNA from the 36 samples was digested with two restriction enzymes (*Pac*I and *Nsi*I), and two indexed adapters were ligated to the digested DNA fragments. Each adapter incorporated a three base pair unique molecular identifier (UMI) region to identify PCR duplicates within each library. The libraries were pooled and aliquoted in three portions to further prevent PCR bias. These aliquots were purified using QIAquick (QIAGEN), size selected for > 150 bp fragments using AMPure XP beads (Beckman Coulter), and nick repaired using DNA polymerase I to repair nicks and improve PCR efficiency. The cleaned libraries were amplified (16 PCR cycles) using KAPA HiFi HotStart ReadyMix (Roche). The PCR reactions were combined, concentrated using QIAquick, size selected again for > 150 bp fragments using AMPure XP beads, and quantified using the KAPA Library Quantification Kit for Illumina platforms (Roche). The final libraries were spiked with 10% PhiX DNA. Sequencing was performed by Novogene (Hongkong) on an Illumina (USA) NovaSeq 6000 platform generating 2 × 150 bp paired-end reads. Raw sequencing reads were deposited in the Sequence Read Archive (SRA) database of the National Center for Biotechnology Information (NCBI) under BioProject accession PRJNA868318.

### GBS data processing

Data analyses were performed on a local Linux cluster node of Radboud University in Nijmegen, The Netherlands, using the scripts provided by Wagemaker et al. [35]. The processing of data followed these main steps: (1) read demultiplexing, adapter removal using AdapterRemoval [56], and merging paired-end reads using Ngmerge [57] with a minimum of 20 bp overlap and a maximum of 10% mismatches (or else joining), (2) meta-reference creation by dereplicating (minuniquesize = 5) and clustering (95% identity) merged monoclonal reads using VSEARCH [58] and filtering non-Eukaryota and Fungi clusters using BLASTN with a minimum alignment length of 40 bp and an e-value of e^−20^, (3) mapping reads from all samples to the meta-reference using STAR [59] allowing multi mapping, (4) removing duplicate reads and reads with low alignment scores (< 0.8), (5) removal of homologous clusters between strains from the meta-reference (see below), calculation of a calibration key from samples with equal diatom proportions, and estimation of relative abundances of the mock mixture samples. Homologous clusters were removed from the meta-reference if (1) more reads mapped to a non-target monoculture cluster (non-target reads > target reads), (2) an insufficient number of reads mapped to a target monoculture cluster (target reads < 8), and (3) the ratio of non-target to target reads of a cluster was below the threshold (non-target/target > 1/15). The filtered meta-reference database file in fasta format from step (2), the mapping file in bam format from step (3), and a stats file in csv format showing the number of reads that mapped to the meta-reference clusters are deposited in the Dryad repository (https://doi.org/10.5061/dryad.gqnk98srr). Python scripts from the msGBS pipeline were used for the steps where no tool is specified. The parameters and more detailed information on the bioinformatic steps can be found in the supporting documents of Wagemaker et al. [35].

### Statistical analyses

Microscope counts of the mock mixtures were compared to LM and GBS-based relative abundance estimates (Additional file 1: Table S7) using a Pearson correlation test and linear regression analysis in R. Regression plots for GBS estimates and pie charts representing community compositions of mock samples were produced using ggplot2 [60].

## Supplementary Information

**Additional file 1: Table S1.** The number of reads and clusters processed during the cluster filtering step of the msGBS pipeline. **Table S2.** Light microscopy counts by the diatom expert based on length, width, and fibula density. **Table S3.** Summary of morphometric measurements in this study compared with earlier measurements. **Table S4.** Light microscopy cell counts of monoclonal samples for the preparation of mixed (mock and calibration) samples. **Table S5.** Calculated volumes (microliters) of monoclonal samples containing the required number of cells for the preparation of mixed (mock and calibration) samples. **Table S6.** Estimated number of cells in calibration and mock samples based on Neubauer counts. **Table S7.** Expected and observed relative abundance estimates for mock samples using GBS and LM methods.

## Data Availability

Raw sequencing reads were deposited in the Sequence Read Archive (SRA) database of the National Center for Biotechnology Information (NCBI) under BioProject accession PRJNA868318. The datasets supporting the conclusions of this article are included within the additional file as supplementary tables. In addition, the filtered meta-reference database file in fasta format, the mapping file in bam format, and a stats file in csv format showing the number of reads that mapped to the meta-reference clusters are publicly available in the Dryad repository [https://doi.org/10.5061/dryad.gqnk98srr].

## Abbreviations

ddPCR: Droplet digital PCR
GBS: Genotyping by sequencing
gDNA: Genomic DNA
HTS: High throughput sequencing
LM: Light microscopy
OTU: Operational taxonomic unit
qPCR: Quantitative PCR
TCC: Thonon culture collection
WFD: Water framework directive
